# A simple non-toxic ethylene carbonate fluorescence in situ hybridization (EC-FISH) for simultaneous detection of repetitive DNA sequences and fluorescent bands in plants

**DOI:** 10.1007/s00709-019-01345-7

**Published:** 2019-01-17

**Authors:** Hieronim Golczyk

**Affiliations:** Department of Molecular Biology, Institute of Biotechnology, John Paul II Catholic University of Lublin, Konstantynów 1i, 20-708 Lublin, Poland

**Keywords:** Chromosome DAPI banding, Ethylene carbonate, Fluorescence in situ hybridization, Heterochromatin, Plants, rDNA

## Abstract

The major drawbacks of standard plant fluorescence in situ hybridization (FISH) designed for double-stranded DNA probes include requirement for experimentally determined heat denaturation of chromosomes at high temperatures and at least overnight hybridization. Consequently, processing with chromosomal preparations may easily result in heat-induced deterioration of chromosomal structural details, is time-consuming, and involves the use of toxic formamide and formaldehyde. Here, I have described a simple and appealing non-toxic procedure with ethylene carbonate (EC)—a formamide-substituting solvent and double-stranded repetitive DNA probes. Applying EC as a component of the hybridization solution at 46 °C not only allowed successful overnight hybridization but also gave a possibility to reduce the hybridization time to 3 h, hence converting the technique into a 1-day procedure. Importantly, the EC-FISH tended to preserve well chromosome structural details, e.g., DAPI-positive bands, thus facilitating simultaneous FISH mapping and chromosome banding on the same slide. The procedure requires no formaldehyde and RNA-se treatment of chromosomes, and no heat denaturation of chromosomal DNA. The key condition is to obtain high-quality cytoplasm-free preparations. The method was reproducible in all the plants studied (*Allium*, *Nigella*, *Tradescantia*, *Vicia*), giving a species-specific signal pattern together with clear DAPI bands on chromosomes. The procedure described here is expected to give a positive stimulus for improving gene-mapping approaches in plants.

**Electronic supplementary material:**

The online version of this article (10.1007/s00709-019-01345-7) contains supplementary material, which is available to authorized users.

## Introduction

Fluorescence in situ hybridization (FISH) with double-stranded DNA probes is a powerful and widely employed technique in biological and medical research. Since the first accommodation to plants (Schwarzacher et al. 1989; Yamamoto and Mukai 1989), its numerous protocols tailored to specific needs have been successfully applied to map DNA sequences physically on plant chromosomes and/or to localize them within cell nuclei or chromatin fibers (e.g., Leitch et al. 1994; Schwarzacher and Heslop-Harrison 2000; Zhang and Friebe 2009; Kato et al. 2011; Walling et al. 2012; Dechyeva and Schmidt 2016; Karafiátová et al. 2016; Badaeva et al. 2017; Singh 2017).

In general, FISH is based on the ability of DNA to undergo a denaturation-renaturation cycle, when after melting DNA, homologous single-stranded sequences find each other and build the double helix during hybridization. The temperatures of DNA water solution for melting (close to 100 °C) and renaturation (60–70 °C), if applied to denaturate chromosomal DNA and for hybridizing it with the probe, would negatively affect chromosome morphology. Therefore, the organic solvent formamide has been widely used as a standard component of hybridization solutions to lower the DNA melting temperature by reducing the thermal stability of the double helix. Consequently, formamide not only lowers the temperature of denaturation but also slows down the rate of renaturation, considerably prolonging the time of hybridization. Typically, a given hybridization solution contains 40–50% formamide, which allows denaturation of chromosomal DNA at around 70–80 °C for several minutes and to run subsequently a relatively stringent—at least overnight hybridization at 37–42 °C (literature cited above). However, several minutes at 70–80 °C is still enough to deteriorate the chromosome structure/morphology. Therefore, to prevent heat-induced chromosomal damage, preparations are fixed with a formaldehyde solution prior to denaturation, while the time and temperature of denaturation and hybridization are experimentally determined. Thus, processing with chromosomal preparations for standard FISH may still easily result in heat-induced deterioration/loss of chromosomal structural details; it is also time-consuming and involves the use of toxic formamide and formaldehyde, i.e., substances that are dangerous to human health. Inhalation and/or skin contact with formamide can cause respiratory tract irritation, headache and nausea, and long-term damage to internal organs, as well potential embryotoxic and teratogenic effects during pregnancy (Sinigaglia et al. 2018, and literature therein). In turn, formaldehyde vapor is known to cause eye and respiration irritation, dermatitis, asthma, pulmonary edema, and respiratory cancer and to accelerate the speed of leukemia development (Norliana et al. 2009; Swenberg et al. 2013).

Recently, a formamide-free FISH technique has been developed for gene aberration tests in human diagnostics, where formamide has been substituted with a non-toxic organic solvent—ethylene carbonate (EC), so that strong signals and low background were obtained in the absence of heat denaturation of both the probe and the target prior to hybridization (Matthiesen and Hansen 2012). In this case, denaturation and hybridization proceeded simultaneously in the same denaturation-hybridization solution—overnight or for 2 h at moderate non-destructive temperatures. Hence, using EC not only allows to drastically shorten the hybridization process and to get rid of formamide, but also potentially may eliminate a need for fomaldehyde fixing of the target.

To my knowledge, there has been no report yet on using EC for FISH in plant kingdom; thus, it is highly desired to test its suitability for plants. The aim of the present paper was to check this possibility, i.e., to develop a simple formamide- and formaldehyde-free plant EC-FISH technique with double-stranded DNA probes. For this purpose, the 5S- and 18S-5.8S-26S rRNA gene arrays were chosen as targets for the detection on chromosomes of several already studied but phylogenetically unrelated plant organisms. The advantage of using 5S rDNA and 18S-5.8S-26S rDNA probes is that tandemly arranged rDNA sequences generate a strong unambiguous chromosomal signal pattern essential for adjusting FISH conditions and are conserved across plant species, while the suitability of other repeats is often limited to single or closely related species. Besides, rRNA gene clusters are by far most widely used and a common starting point for FISH mapping or karyotyping (Figueroa et al. 2012 and literature therein).

All the plant species tested here have well-documented constitutive heterochromatin on their chromosomes (see “Results and discussion”), which generally tends to form DAPI-positive bands when subjected to differential fluorescence treatments. Since simultaneous FISH mapping and well-preserved banding on the same preparation in plants is rather uncommon when standard formamide-based FISH techniques are applied, the latter seems to interfere with the differential DAPI fluorescence. Hence, there was a hope that the novel formamide-free EC-FISH plant technique would yield clearly visible chromosome bands, which could be observed along with FISH signals on the same preparation. Indeed, this was fully achieved. The protocol and observations described here are expected to give a positive stimulus for improving gene-mapping approaches in plants.

## Materials and methods

### Plant material and chromosome preparations

The starting plant material consisted of commercially available seeds of *Nigella damascena*, *Vicia faba*, and bulbs of *Allium cepa*, as well as stem cuttings of *Tradescantia spathacea* (syn. *Rhoeo spathacea*), the latter derived from own stock. The seeds of *N. damascene* and *V. faba* were germinated on wet filter paper in Petri dishes at 25 °C in the dark. The *A. cepa* bulbs and *T. spathacea* stem cuttings were grown in tap water in glass jars wrapped with aluminum foil at 25–28 °C.

Root tips excised from actively growing roots of all plants were harvested, pretreated with a saturated aqueous solution of α-bromonaphtalene at room temperature (RT) for 4 h, fixed in freshly made AA fixative (3:1 absolute ethanol: glacial acetic acid mixture) at RT overnight, and after replacement of the fixative with a fresh one, stored at − 20 °C until use. Fixed root tips were washed in 0.01 M citric buffer pH 4.6–4.8 at RT for 3 × 10 min and then macerated at 37 °C for 15 min–2 h (depending on the species and/or root tip size) in a mixture of 20% (*v*/*v*) pectinase (Sigma), 1% (*w*/w*)* cellulase (Sigma-Aldrich), and 2% (*w*/*w*) cellulase Onozuka RS (Serva) in 0.01 M citric buffer at pH 4.6–4.8. The material was washed in the same citric buffer at RT for 3 × 10 min and then incubated in 60% acetic acid for 15 min–2 h at RT. The macerated tissues starting to become tissue suspensions were carefully transferred together with some amount of acetic acid onto the Superfrost microscope slides (Menzel) using a Pasteur pipette. The tissue suspension on the slide was thoroughly mixed with a needle, the material was covered with a coverslip, gently thumb-pressed, and quickly flame-heated with an alcohol burner. The preparations were further macerated by keeping on a hot plate at 75 °C for 2–10 min while some 60% acetic acid was being added to the coverslip edges to prevent evaporation. Then a tapping with a thin wooden stick was applied to the preparations, which were then squeezed by thumb-pressing and frozen in liquid nitrogen. After detaching of the coverslips, the slides were air-dried and aged at RT for 1 to 2 days.

### Slide pretreatment

The pretreatments of preparations for both standard FISH and EC-FISH proceeded as follows*:* (a) incubation in an aqueous solution of 0.01 N HCl at 37 °C for 10 min; (b) digestion in a pepsin (Sigma-Aldrich) work solution (0.25 mg/ml) in 0.01 N HCl at 37 °C for 15 min to 1 h (preparations inspected under a phase contrast microscope); (c) washing 6 × 5 min in 0.01 N HCl at 37 °C; (d) draining of the slides on a paper towel and incubation in 0.5 N HCl for 5–15 min at RT; (e) washing in distilled water for 2 × 5 min at RT and brief draining; (f) incubation in freshly made AA for 10 min at RT; (g) air-drying and storing in hermetic plastic boxes at 4 °C until use.

Preparations destined for standard FISH were further treated as follows: (h) incubation in 200 μl of RNase A (Sigma-Aldrich) 100 μg/ml work solution in 2 × SSC (saline sodium citrate: 0.3 M sodium chloride + 0.03 M trisodium citrate; pH 7.0) at 37 °C for 1 h; (i) washing the slides three times for 5 min each in 2 × SSC at RT; (j) incubation in 1× PBS (phosphate-buffered saline pH 7.0) for 10 min at RT; (k) fixing in a ca. 3.6% freshly prepared formaldehyde solution (formalin diluted ten times) in 1× PBS for 10 min at RT; (l) washing 3 × 5 min in 1× PBS at RT; (m) washing in distilled water 2 × 5 min at RT and draining; (n) incubation in freshly made AA for 10 min at RT, and air-drying. The air-dried preparations, if not used on the same day, were stored at 4 °C until required.

### Molecular probes for standard FISH and EC-FISH

To detect evolutionarily conserved 18S-5.8S-26S and 5S rDNA sites, a 2.3 kb ClaI fragment of the 26S rDNA ribosomal gene of *A. thaliana* (Unfried and Gruendler 1990) labeled with digoxigenin-11-dUTP (Roche Applied Science) by nick translation and the 5S rDNA coding unit, PCR-labeled with tetramethyl-rhodamine-5-dUTP (Roche Applied Science), were obtained as previously described (Golczyk 2011a; Golczyk et al. 2014) and used as probes.

### Denaturation/hybridization and stringent washes for the standard FISH

The hybridization mix for standard FISH consisted of 10% (*w*/*v*) dextran sulfate (Sigma-Aldrich), 45% (*v*/*v*) formamide (Sigma Aldrich), 2–3 ng/μl of each of the two labeled rDNA probes, and saline sodium citrate at 2× final concentration (0.3 M sodium chloride, 0.03 M trisodium citrate). For one hybridization reaction, 40 μl of this mix was set up in a 200-μl PCR tube, incubated at 90 °C for 4 min in a thermocycler, immediately cooled on ice for 10 min, mounted onto a preparation, and covered with a coverslip with subsequent sealing of the coverslip edges with Rubber cement (Marabu). The preparations together with the probes were then denatured on a hot plate at 77 °C for 5 min and then hybridized at 37 °C overnight in a humid plastic box. Stringent washes were performed twice in 15% (*v*/*v*) formamide in 0.1 × SSC for 5 min at 42 °C. Then, the slides were washed twice in 2 × SSC for 3 min at 42 °C and twice in 2 × SSC for 3 min at RT before they were subjected to signal detection.

### Denaturation/hybridization and stringent washes for the EC-FISH

The hybridization mix for EC-FISH consisted of 15% (*v*/*v*) melted ethylene carbonate (Sigma-Aldrich), 10% dextran sulfate (Sigma-Aldrich), 0.6 M sodium chloride, 0.01 M trisodium citrate, and 2–3 ng/μl of each of the two labeled rDNA probes. For one hybridization reaction, 50 μl of this mix was set up in a 200-μl PCR tube, incubated at 90 °C for 4 min in a thermocycler, and immediately cooled on ice for 10 min. Hybridization mixes prepared at RT (incubation at 90 °C omitted) were also tested. The preparations were incubated in the hybridizing mix covered with a plastic coverslip in a humid plastic box at 46 °C or at 50 °C for 3 h or overnight. Stringent washes were performed 2 × 3 min in 2 × SSC at 50 °C or at 55 °C. Then, the slides were washed twice in 2 × SSC for 5 min at RT before they were subjected to signal detection.

### Signal detection and image acquisition/processing for standard FISH and EC-FISH

The preparations were transferred from 2 × SSC to 0.1% (*v*/*v*) Tween (Sigma Aldrich) in 4 × SSC (4 × SSC/Tween) and kept there at RT for several minutes. After a quick draining of the slides on a paper towel, 100 μl of anti-digoxigenin-FITC (Roche Applied Science) diluted 1:70 in 4 × SSC/Tween +3% (*w*/*v*) BSA (Bovine Serum Albumine; Sigma-Aldrich) was mounted on a slide, covered with a plastic coverslip and incubated for 1 h at 37 °C in the humid plastic box. Then the preparations were washed three times in 4 × SSC/Tween (7 min each wash) at 37 °C (water bath), drained and mounted in Vectashield antifade medium (Vector Laboratories) supplemented with DAPI (4′,6-diamidino-2-phenylindole, Sigma-Aldrich) at 1 μg/μl final concentration. The preparations were analyzed under 100× oil immersion objective of the Nikon NiU epifluorescent microscope and images were captured with a cooled monochrome DSQi1 camera (Nikon) to the computer using NIS Elements software (Laboratory Imaging, Ltd.) and uniformly processed in Adobe Photoshop CS3 (Adobe Systems). Additionally, to confirm the assignment of a correct number to a given chromosome pair (within the metaphase plate), chromosome measurements were carried out on some digitally captured metaphases using the public domain Fiji software package (https://fiji.sc/).

## Results and discussion

From a historical perspective, FISH technology used in plants almost always appears to lag behind the one applied in humans and animals. Likewise, in human/animal molecular cytogenetics, ethylene carbonate (EC) is now quite frequently used as a component of formamide-free hybridization mixes (e.g., Matthiesen and Hansen 2012; Linhoff et al. 2015; Shigeto et al. 2016); yet, to my knowledge, there has been no report on application thereof in plants. Other formamide-free FISH protocols with double-stranded DNA probes developed for plants (e.g., Kato et al. 2004; Chester et al. 2012; Jang and Weiss-Schneeweiss 2015) did not involve EC but included heat denaturation of preparations prior to hybridization with mostly overnight hybridization; thus, the techniques were actually quite similar to the standard FISH procedure.

**Fig. 1:**
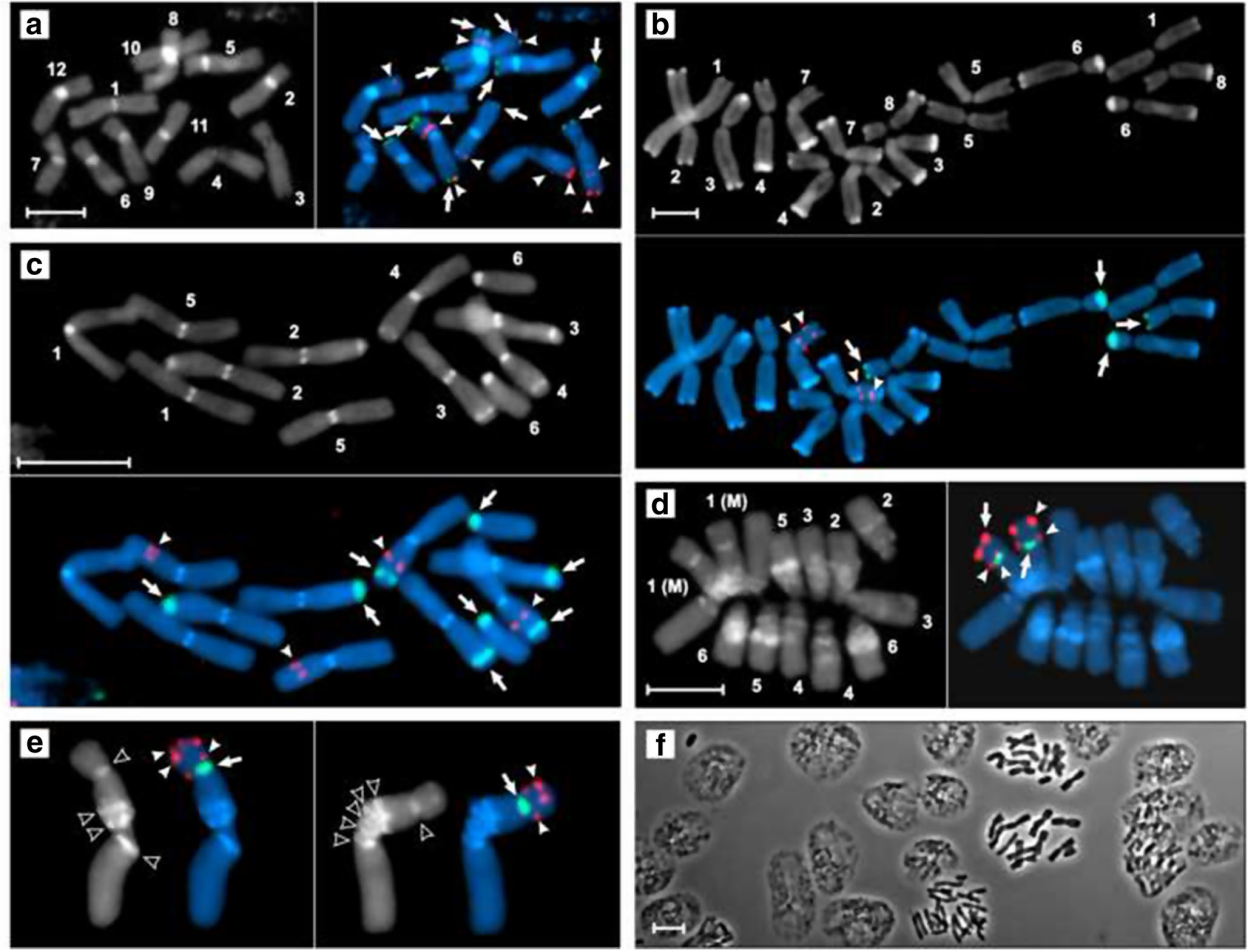
**a**–**e** Simultaneous DAPI-banding (gray scale images) and EC-FISH (color images) of the two rDNA probes—5S rDNA (red signals and solid arrowheads) and 26S rDNA (green signals and solid arrows) to the somatic metaphase chromosomes of *T. spathacea* (**a**), *A. cepa* (**b**), *N. damascena* (**c**), and *V. faba* (**d**–**e**). Open arrowheads point to heterochromatic DAPI bands of M chromosome in *V. faba*. Chromosome pairs identified and numbered; **f** a fragment of the cytoplasm-free preparation of *N. damacsena* viewed under a phase contrast microscope. Bars = 10 μm

In spite of the theoretical assumptions on DNA-DNA interactions derived from molecular biology, there is no strict common recipe for hybridization and posthybridization conditions that could be equally optimal for diverse cytogenetic contexts. They are still the matter of a trial and error optimization (Fontenete et al. 2016). With respect to the signal strength and consistency in the arrangement of rDNA loci, the EC-FISH technique gave the same response to all the described hybridizing and posthybridizing conditions (see “Materials and methods”) in each of the tested species (Fig. 1a–e). Importantly, the same quality and pattern of hybridization signals was obtained with the standard formamide FISH technique (“Electronic supplementary material,” Figs. S1a-d). The results concerning *T. spathacea* also strongly highlight that the chosen conditions of EC-FISH are within the optimal range. This species is a permanent translocation heterozygote deprived of regular chromosome pairs and with numerous rDNA loci whose arrangement suggests complex chromosome rearrangements (Golczyk 2013). To identify its chromosomes correctly, a sensitive double target in situ hybridization with the two rDNA probes is needed (Golczyk et al. 2005, 2010). Its twelve chromosomes harbor ten 5S rDNA loci and ten 18S-5.8S-26S rDNA clusters on their arms, some of them small and hardly detectable, e.g., 5S rDNA intercalary loci on chromosomes 3 and 4 (Golczyk et al. 2010). Here, all these rDNA loci were revealed easily and unambiguously with the EC-FISH technique (Fig. 1a). Moreover, the species possesses also some extra 18S-5.8S-26S rDNA remnants dispersed throughout the heterochromatic pericentromeres of all the twelve chromosomes. The pericentromeric rDNA signals on mitotic chromosomes are not detectable with the standard high-stringency FISH; however, they can be revealed when lowering the FISH stringency (Golczyk et al. 2010). Since the pericentromeric rDNA was not detectable with the EC-FISH technique (Fig. 1a), another proof is at hand to regard the chosen conditions as the optimal and stringent ones. Thus, there was no need for testing incubations other than these described in the “Materials and methods” section. The detected arrangement of rDNA loci in *A. cepa* (Fig. 1b), *N. damascena* (Fig. 1c), and *V. faba* (Fig. 1d–e) conformed to the rDNA chromosomal pattern already described by other authors (Hizume 1992, 1993; Shibata and Hizume 2002; Raina et al. 2010; Hizume et al. 2013). The novel findings include (i) the 18S-5.8S-26S rDNA locus of chromosome pair 3 in *N. damascena* occupies intercalary position (Fig. 1c); (ii) as a result of obtaining superior preparations, it was revealed that the big subterminal 5S rDNA locus on the nucleolar arm of chromosome M in *V. faba* (Fig. 1d) actually consisted of two smaller adjacent loci (Fig. 1e).

The EC-FISH technique described here does not need toxic formaldehyde and formamide and requires no RNA-se treatment of chromosomes, no chromosome/probe heat denaturation, and can be shortened to a 1-day procedure with 3 h of hybridization at 46 °C or 50 °C if required (see “Materials and methods”). Matthiesen and Hansen (2012) demonstrated that 15% EC present in the hybridization solution containing 0.6 M sodium chloride substantially increased the hybridization rate at a lower denaturation and hybridization temperature with the resultant strong FISH signals and reduced background staining. This finally allowed them to optimize 2-h hybridization at 50 °C as well as the overnight hybridization at 45 °C, both generating satisfying results. Among the protocol settings used for EC-FISH (see “Materials and methods”), the mildest conditions, i.e., hybridization at 46 °C for 3 h or overnight followed by washing in 2 × SSC at 50 °C seem to be the best balanced option for obtaining clear hybridization signals along with satisfying sharpness of DAPI bands and a good overall chromosome morphology (see below). However, the most critical, both for EC-FISH and standard FISH, was to obtain high-quality cytoplasm-free preparations selected under the phase contrast microscope (Fig. 1f). That is why proper enzymatic digestion of root meristems, prolonged incubation thereof in 60% acetic acid, and further pepsin treatment are the steps with the most beneficial effects. If even little cytoplasm was present, hybridization signals were weak or absent. In contrast, incubation in the RNase A solution included in many protocols was unnecessary and could be skipped. Although RNase treatment is routinely applied for standard FISH, actually it is optional and can be omitted (Badaeva et al. 2017; Liehr et al. 2017).

All the species tested here have well-documented arrangement of constitutive heterochromatin on their chromosomes (Kalkman 1984; Klásterská and Natarajan 1975; Pich et al. 1996; Golczyk et al. 2010; Golczyk 2011b). Since plant DAPI bands generally tend to conform to heterochromatin (Barros e Silva and Guerra 2010 and literature therein), there was a hope that the EC-FISH technique under the conditions described above would preserve this pattern well in the form of clearly visible DAPI bands observed along the FISH signals in the same preparation. Indeed, it was fully realized here. All the protocol settings for the EC-FISH (see “Materials and methods”) generated the DAPI bands sharply delimited from the rest of chromatin (Fig. 1a–e). These bands represented pericentromeric heterochromatin of *T. spathacea* (Fig. 1a), telomeric heterochromatin of *A. cepa* (Fig. 1b), centromeric and intercalary heterochromatin of *N. damascena* (Fig. 1c), and pericentromeric and interstitial heterochromatin of *V. faba* (Fig. 1d, e). The AT-rich pericentromeric heterochromatin forms massive blocks on ten *T. spathacea* chromosomes, but merely tiny centromeric dots can be detected on chromosomes 3 and 4 (Golczyk et al. 2010). This specific DAPI fluorescence pattern on somatic chromosomes has been so far best documented with DAPI/actinomycin D (DAPI/AMD) base-specific fluorescence (Golczyk et al. 2010). To note, the quality of the DAPI bands generated by EC-FISH in this species (Fig. 1a) is essentially the same as of those obtained with the use of the DAPI/AMD technique. In *A. cepa*, all chromosome ends are equipped with terminal heterochromatin (Kalkman 1984; Pich et al. 1996), which is DAPI-positive, as shown clearly in Fig. 1b. The size of the terminal DAPI bands varied between homologous pairs and, together with the FISH signals and chromosome morphology, was a good basis for identification of pairs: 1, 2, 6, 7, and 8. Assigning correct numbers to the other pairs (3, 4, 5) according to the existing convention (De Vries 1990 and literature therein) required however chromosome measurements (chromosome length and arm ratio). The arrangement of DAPI bands in *N. damascena* karyotype (Fig. 1c) strictly mirrored the heterochromatin pattern described by Klásterská and Natarajan (1975), who applied the SSC-Giemsa C-banding technique. It shows however more DAPI bands than the fluorescent DAPI-banding karyotype of Hizume et al. (1989). Due to the successful simultaneous combination of the DAPI differential fluorescence with the FISH signals within the same preparation, it is evident that four 18S-5.8S-26S rRNA gene clusters on chromosome pairs 2 and 3 are components of the intercalary heterochromatic bands (Fig. 1c). *Vicia faba* chromosomes have emerged as a classical object for testing cytogenetic techniques since the pioneering work of Caspersson et al. (1969). Especially the large nucleolar chromosomes of the first pair, i.e., M chromosomes, the only metacentrics chromosomes in the karyotype, which exhibit a complex banding pattern around their centromeres. From two to five heterochromatin bands were reported by different authors in this region, depending on the technique used and on the degree of chromosome contraction (e.g., Döbel et al. 1973; Greilhuber 1975; Klásterská and Natarajan 1975; Rowland 1981 and others cited therein). The highest C-banding resolution in this region was obtained by Greilhuber (1975) on extended prophase chromosomes, i.e., four bands on the nucleolar arm and one band on the other arm. The *V. faba* somatic chromosomes studied here were highly condensed (Fig. 1d, e); yet, the obtained banding pattern allowed distinguishing chromosome pairs (Fig. 1d) according to the karyotyping established by the authors cited above. Moreover, from three to five distinct DAPI bands were observed around the centromere of M chromosomes (Fig. 1e). This means that the EC-FISH technique appears a promising tool for combining FISH signals with a satisfying DAPI-banding resolution—even on highly condensed mitotic chromosomes.

Chromosome bands are higher-order genomic structures with epigenetic flavors, important to understand the functional aspects of genome organization (Holmquist 1992). They are usually not uniform in terms of sequence composition; thus, the information whether a given sequence belongs to a given band or how it is positioned in relation to the band is of prime interest (Robledillo et al. 2018). Typically, to deduce the position of FISH signals in relation to banding, fluorescent differential staining and FISH are done separately on different slides or infrequently—sequentially on the same slide. In the latter case, after subjecting to a fluorescent differential staining, the preparation is then destained and treated according to the FISH protocol (e.g., Golczyk et al. 2010; Ansari et al. 2016). The disadvantage of the sequential procedures is however that previously identified and photographed chromosomes/nuclei, when further processed for FISH (detaching of the coverslip, removing the mounting medium from the slide, destaining and in situ hybridization procedure), are difficult to find, fragmented, lost, and/or changing their position on the slide, which renders such a highly troublesome and tricky mapping extremely difficult.

In contrast, the standard in situ procedure resulted in (*i*) bands in *T. spathacea* that were not as clear as those generated by EC-FISH/DAPI or in (*ii*) very poorly developed or lacking fluorescent longitudinal differentiation on chromosomes of *A. cepa*, *N. damascena*, and *V. faba* (“Electronic supplementary material,” Fig. S1a-d). This indicates that standard in situ hybridization protocol interferes with differential DAPI fluorescence. Indeed, the simultaneous FISH mapping and well-preserved chromosome banding on the same preparation, as was done here, is rather uncommon in plant cytogenetics.

In conclusion, the proposed here plant EC-FISH technique appears highly attractive, since it requires no formaldehyde and RNA-se treatment of chromosomes and does not need formamide, heat denaturation of chromosomal DNA for successful hybridization/denaturation. Notably, it can be shortened to a 1-day procedure with 3 h of hybridization at moderate temperature. Moreover, it preserves clearly sharp DAPI bands simultaneously with the FISH signals within the same preparation. Thus, the procedure is expected to give a positive stimulus for improving gene-mapping approaches in plants as well as chromosome identification and karyotyping. Further development of the plant EC-FISH technique in diverse cytogenetic contexts is anticipated.

## Electronic supplementary material

Fig. S1 DAPI-staining (left images) and standard FISH (right images) of the two rDNA probes - 5S rDNA (red signals and solid arrowheads) and 26S rDNA (green signals and solid arrows) to the somatic metaphase chromosomes of *T. spathacea* (**a**), *A. cepa* (**b**), *N. damascena* (**c**), *V. faba* (**d**). Only chomosomes 3 and 4 of *T. spathacea* were numbered. Open arrowheads point to the best visible heterochromatic DAPI-bands. Bars = 10 μm. (PNG 2765 kb)

High Resolution Image (TIF 11205 kb)

## Abbreviations

DAPI: 4′,6-Diamidino-2-phenylindole
EC: Ethylene carbonate
EC-FISH: Ethylene carbonate fluorescence in situ hybridization
FISH: Fluorescence in situ hybridization
rDNA: Ribosomal DNA
