# Supplementary figures and images for: A simple non-toxic ethylene carbonate fluorescence in situ hybridization (EC-FISH) for simultaneous detection of repetitive DNA sequences and fluorescent bands in plants
Source: Protoplasma. 2019 Jan 17;256(3):873–80. doi: 10.1007/s00709-019-01345-7 (PMC6482133; doi:10.1007/s00709-019-01345-7)

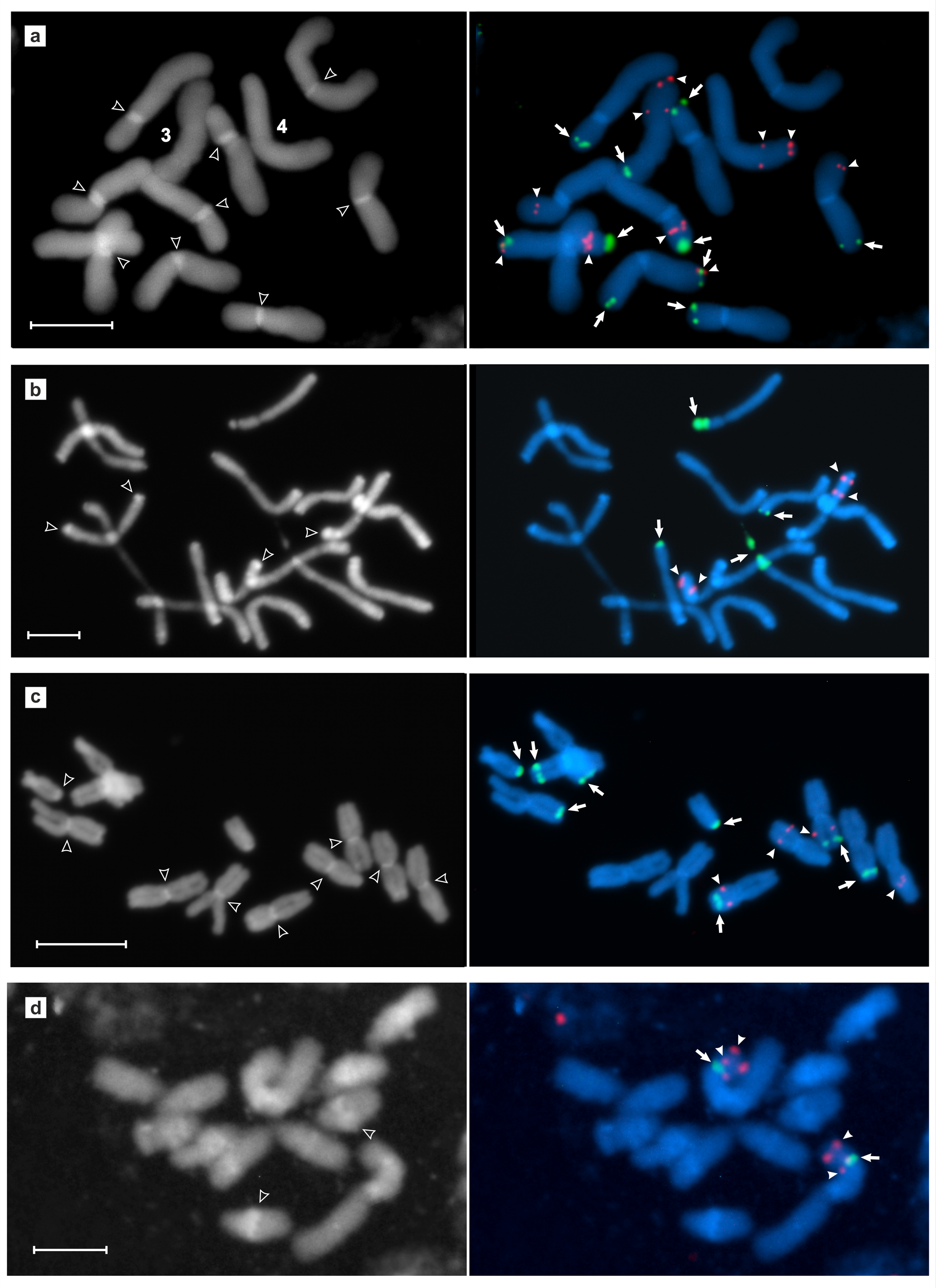

Supplement: Supplementary file 1 — DAPI-staining (left images) and standard FISH (right images) of the two rDNA probes - 5S rDNA (red signals and solid arrowheads) and 26S rDNA (green signals and solid arrows) to the somatic metaphase chromosomes of T. spathacea (a), A. cepa (b), N. damascena (c), V. faba (d). Only chomosomes 3 and 4 of T. spathacea were numbered. Open arrowheads point to the best visible heterochromatic DAPI-bands. Bars = 10 μm. (PNG 2765 kb) [file 709_2019_1345_Fig2_ESM.png]

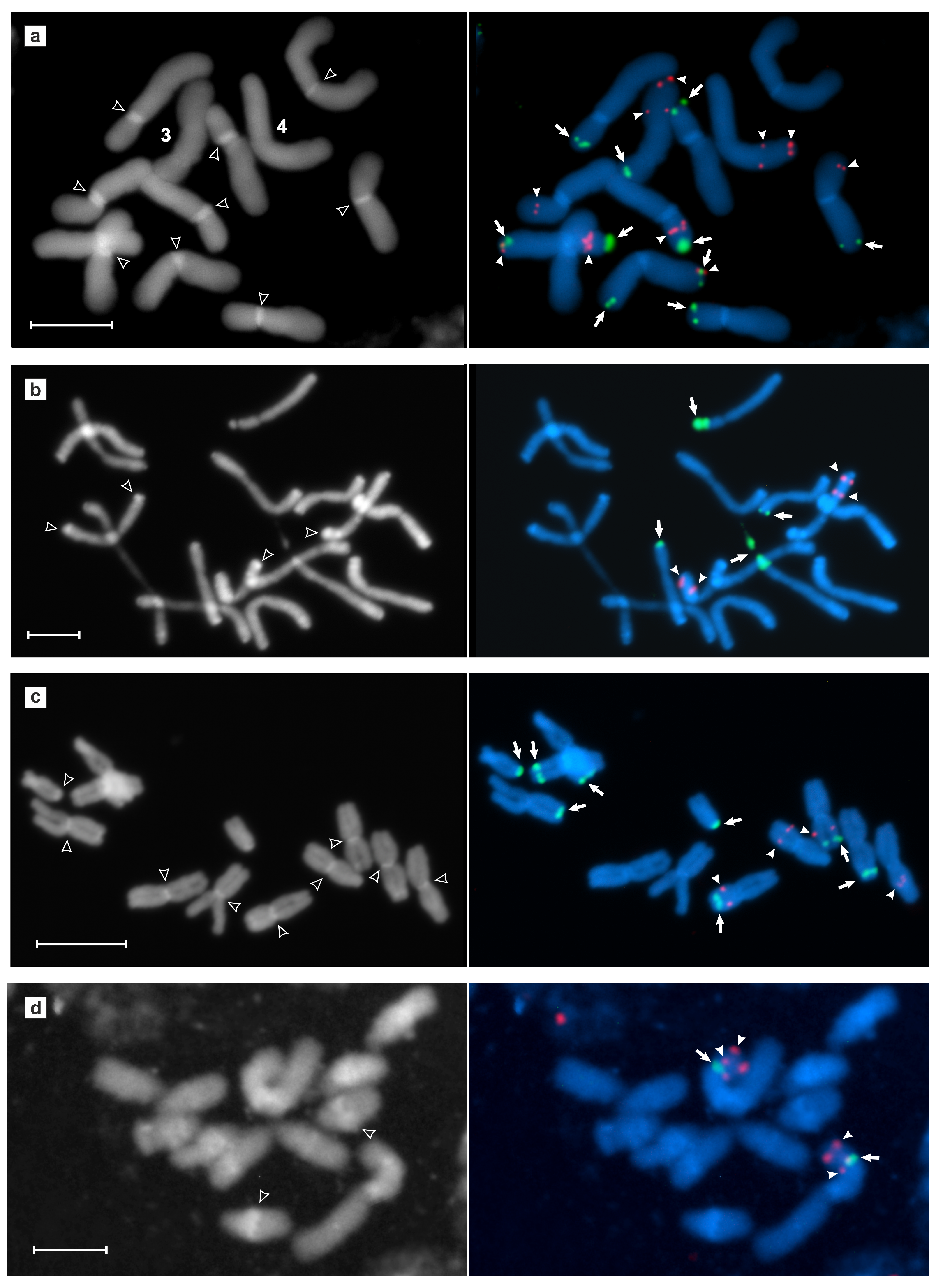

Supplement: Supplementary file 2 — High Resolution Image (TIF 11205 kb) [file 709_2019_1345_MOESM1_ESM.tif]
